# Limited evidence for alterations in Gag-mediated HIV replication capacity over the course of the North American epidemic (1979-present)

**DOI:** 10.1186/1742-4690-9-S2-P157

**Published:** 2012-09-13

**Authors:** L Cotton, D Chopera, K Penney, J Carlson, E Martin, A Le, T Kuang, B Walker, J Fuchs, S Buchbinder, T Wagner, M John, S Mallal, B Koblin, K Mayer, A Poon, M Brockman, Z Brumme

**Affiliations:** 1 Simon Fraser University, Burnaby, Canada; 2 Microsoft Research, Los Angeles, CA, USA; 3 Ragon Institute, Charlestown, MA, USA; 4 San Francisco Dept of Public Health, San Francisco, CA, USA; 5 Murdoch University, Perth, Australia; 6 New York Blood Center, New York, NY, USA; 7 Fenway Community Health, Boston, MA, USA; 8 BC Centre for Excellence in HIV/AIDS, Vancouver, Canada

## Background

The extent to which HIV replication capacity (RC) has changed over the epidemic’s course, and the influence of HLA-associated immune pressure as its driving force remains unknown. We performed a comparative study of immune escape and RC in historic (1979-1989) and modern Gag subtype B sequences from North America.

## Methods

Using phylogenetically-informed methods, we identified HLA-associated Gag polymorphisms in a historic cohort (N=239; 1979-1989). We also generated recombinant NL4-3 viruses encoding clonal plasma RNA Gag from 80 historic and 58 modern (2002-2008) sequences. Viral RC was measured using a GFP reporter T-cell assay and results were normalized to NL4-3 controls.

## Results

95% of HLA-associated polymorphisms identified in the historic cohort were consistent with published modern escape pathways. Overall, the prevalence of HLA-associated polymorphisms in the general population increased a median 1.3-fold between historic and modern sequences; however in many cases this was influenced by differences in HLA allele frequencies between HIV-infected populations examined. Of note, the prevalence of the B*27-associated R264K escape mutation increased from 0.4 to 1.3% in the general population over time despite B*27 allele frequency remaining constant at 2.5%. Modestly lower viral RC was observed for Gag recombinant viruses constructed from pre-1985 sequences (median 0.86 [IQR 0.78-0.97], N=24) compared to those from 1985-1989 (median 0.98 [IQR 0.87-1.05], N=56) and 2002-2008 (median 0.96 [IQR 0.83-0.1.10], N=58) (p=0.049). In both historic and modern cohorts, host expression of HLA-B*27 was associated with lower RC (p=0.007). Gag codons associated with lower RC, including S67A, were identified in an exploratory analysis.

## Conclusion

Gag-mediated viral RC may have increased modestly since the beginning of the North American epidemic, despite limited evidence for HLA-driven viral sequence evolution during this time. Although mechanisms driving RC differences remain unclear, results do not support rapid and substantial accumulation of HLA-driven escape mutations in circulating North American HIV-1 sequences.

